# New Insights in the Diagnosis and Treatment of Heart Failure

**DOI:** 10.1155/2015/265260

**Published:** 2015-10-26

**Authors:** Giulio Agnetti, Massimo F. Piepoli, Giuseppe Siniscalchi, Francesco Nicolini

**Affiliations:** ^1^Division of Cardiology, Johns Hopkins University School of Medicine, Baltimore, MD 21205, USA; ^2^DIBINEM, University of Bologna, 40126 Bologna, Italy; ^3^Heart Failure Unit, Cardiology Department, Guglielmo da Saliceto Hospital, 29121 Piacenza, Italy; ^4^Department of Cardiovascular Surgery, University Hospital Lausanne, 1011 Lausanne, Switzerland; ^5^Cardiac Surgery Unit, Department of Clinical and Experimental Medicine, University of Parma, Via A. Gramsci 14, 43126 Parma, Italy

## Abstract

Cardiovascular disease is the leading cause of mortality in the US and in westernized countries with ischemic heart disease accounting for the majority of these deaths. Paradoxically, the improvements in the medical and surgical treatments of acute coronary syndrome are leading to an increasing number of “survivors” who are then developing heart failure. Despite considerable advances in its management, the gold standard for the treatment of end-stage heart failure patients remains heart transplantation. Nevertheless, this procedure can be offered only to a small percentage of patients who could benefit from a new heart due to the limited availability of donor organs. The aim of this review is to evaluate the safety and efficacy of innovative approaches in the diagnosis and treatment of patients refractory to standard medical therapy and excluded from cardiac transplantation lists.

## 1. Introduction

Cardiovascular disease is the leading cause of mortality in USA and Western countries with ischemic heart disease accounting for the majority of these deaths. Paradoxically, the improvements in the medical and surgical treatment of acute coronary syndromes are leading to an increasing number of “survivors” who are then developing heart failure. Despite considerable advances in the management of heart failure, the gold standard for the treatment of end-stage heart failure patients remains heart transplantation. Nevertheless, this procedure can be offered only to a small percentage of patients who could benefit from a new heart due to the limited availability of donor organs. In fact, the number of heart transplants has remained static worldwide and the number of heart transplants performed each year in the US has plateaued at about 2100 for the past few years. Improving awareness of the very end stages of heart failure is emerging as a major need for the clinical community, and implementing best practices for palliative care is also imperative.

A number of innovative approaches are being investigated on the basis of improved survival and quality of life in patients refractory to medical therapy and excluded from cardiac transplantation lists. These procedures include the optimization of medical therapy, coronary artery bypass surgery and valve surgery in high risk patients, ventricular restoration techniques, and the implantation of ventricular assist devices as destination therapy or other approaches (such as cardiac resynchronization therapy) [1]. Future therapies for heart failure could include new approaches with stem cell therapy, associated with standard revascularization techniques or with other procedures such as the implantation of innovative ventricular assist devices, new ventricular restoration techniques, or new drugs.

The continuous innovations in proteomic technologies will help pinpoint protein posttranslational modifications that could help elucidate the transition to heart failure (HF). This link between biology and technology could greatly assist in identifying biomarkers with increased specificity as well as more effective therapies.

## 2. Proteomics to Understand Heart Failure

### 2.1. The Contribution of Proteomics to Our Understanding of Biological Systems

Nowadays, mass spectrometry (MS) is used to detect, identify, and quantify a wide array of compounds spanning from small molecules, pharmaceuticals, metabolites (hence metabolomics), lipids (hence lipidomics), and peptides and proteins (hence peptidomics and proteomics). In the last four, the “-omics” suffix implies that hundreds to thousands of compounds can be detected in a single analysis providing a snapshot of a given metabolome, lipidome, peptidome, or proteome, respectively. As it is easy to imagine this capability has enhanced tremendously our understanding of biological systems. For the sake of brevity we will address the contribution of proteomics to HF research in this section. For the same reason we cannot be exhaustive and defer to other comprehensive reviews on cardiovascular proteomics for the interested reader [2].

The proteome was first defined publicly a little over a decade ago as the “protein complement of the genome” or the protein make-up that can be identified and quantified from a given biological sample. As an axiom, proteomics is the complex of technologies (centered around MS) used to study the proteome. Perhaps the most important contribution of these technologies to modern medicine is the discovery of the dazzling diversity of protein posttranslational modifications (PTMs). There are over 400 PTMs, such as phosphorylation, nitrosylation, acetylation, and methylation, currently listed in protein databases [3]. The vast majority of PTMs have an effect on a protein's life may it be activity, localization, turnover, and so forth or in other words its function. Posttranslational modifications are the most likely integrators of the interactions between the phenotype and the environment due to their dynamic regulation and this new knowledge has profound implications for biomedicine. For instance, the sporadic nature of many diseases, such as HF, could be explained in the light of proteins and their PTMs rather than the genetic background. In fact, the prediction of a phenotype solely based on genes is inherently complicated by the exponential increase in complexity when moving genes through transcripts to modified proteins and their complexes. The realization that PTMs are so abundant in nature is daunting; however, the technological advances seen in the last decade let us hope that their mapping is within reach and that with this information we will have a high-resolution picture of the molecular phenotype of many diseases in the near future.

As technologies quickly develop, their potential clinical applications also multiply. Like the computer industry some of these technologies, and mainly MS, have now reached a point where performance has allowed targeting an intermediate segment of the users market. That is to say that high-performance MS instruments which were previously relegated to well-funded and highly specialized research groups are now slowly becoming accessible to smaller institutions, including hospitals and clinical labs. The great potentials for biomarkers discovery and clinical labs analyses are still largely unmet by the limited knowledge of the scientific and medical communities.

### 2.2. A Brief Overview of the Technical Aspects of Proteomics

Mass spectrometers are classically named after their anatomy and are composed of a source, one or more analyzers, and a detector. For instance, a matrix assisted laser desorption ionization (MALDI) is a type of source, whereas time-of-flight (TOF) is one of the first used analyzers. The source is the part of the instrument where analytes (e.g., peptides and proteins in proteomics) are ionized so that they can be separated according to their mass (mass/charge or *m*/*z*) in the analyzer. Most commonly MS are coupled to liquid chromatography (LC, hence LC-MS). However, MS that are coupled with an LC typically have different sources than MALDI (such as Electron Spray Ionization or ESI) and analyzers (such as quadrupoles or “Q” and ion traps). To complicate things further, most modern instruments have multiple analyzers in series (hence Q-trap, Q-TOF, triple-Q, etc.). These last instruments are also referred to as tandem MS (or MS/MS) and the advantage of having multiple analyzers resides in the capability of sequencing a peptide (and often assigning PTMs unambiguously), with the cost being the time for acquisition (or analysis). The number of methodological approaches that have arisen in the last decade is also complex. They can be broadly divided into protein- and peptide-centric (or top-down and bottom-up to use a widespread nomenclature, resp.). The most common approaches are peptide-centric, which means that proteins are digested into peptides prior to MS analysis due to the increased stability of the latters and the fact that they can be measured more accurately. The separation of proteins prior to MS analysis can be achieved by polyacrylamide gel electrophoresis (PAGE) or LC (hence gel-based and gel-free approaches); however, LC is also used to inject proteins and peptides directly in the MS. Moreover, other separation techniques such as capillary electrophoresis (CE) can be also utilized [4]. One of the typical approaches based on the direct LC-MS analysis of digested proteomes is commonly known as “shotgun” [5], as peptides are digested, desalted, and injected into the MS. When it comes to quantification, two different schools of thought advocate for label and label-free approaches. In the former, peptides are chemically derived with various chemical “tags” prior to MS analysis. These are released in the MS to work as “reporters” for the quantity of a given peptide (and therefore protein) [6]. However, due to the increased reproducibility of separation and MS technologies, it is now possible to have an accurate quantification also in absence of reporters (label-free) [4]. Finally, the clinical relevance of top-down or protein-centric proteomics in HF research is also rapidly emerging [7]. Peptide-centric approaches can be utilized for both the “entire” proteome (proteome-wide) or fractions of it (subproteomes). Indeed the complexity of biological systems is such that it is hard to predict when full proteome-wide coverage will be achieved for complex samples. The detection of peptides in a MS is a competitive process; therefore the higher the complexity of the sample, the higher the chance that low-abundant peptides (proteins) may be missed. For this reason, the enrichment of specific PTMs (e.g., phosphoproteome [6]) or subproteomes (e.g., different organelles [8]) greatly enhances sensitivity. Targeted proteomics or the application of these technologies to highly enriched subproteomes (e.g., individual proteins end, their PTMs, and their complexes) is arguably the best approach to gain the deepest level of detail. A successful example of this concept is the crossover of a MS technique known as multiple reaction monitoring (MRM) from the pharmaceutical industry to proteomics. Briefly MRM allows to precisely quantify proteins using the quantity of few peptide fragments in a tandem MS. The use of isotopically labeled internal standard enables absolute quantification. As an example, multiple reaction monitoring was recently used to accurately quantify the phosphorylation sites (known and new) of cardiac TnI, one of the gold standard markers to diagnose cardiac ischemia [9].

### 2.3. Proteomics to Tackle Emerging Concepts in HF Research

There has been an increasing consensus on the similarities between well-established organ proteinopathies (such as Alzheimer's and Parkinson's diseases) and HF [10]. This concept was pioneered a little over ten years ago by Robbins and colleagues who reported the presence of preamyloid oligomers (PAOs) similar to those observed in the brains of Alzheimer's patients, in cardiac specimens from HF patients [11]. In the last few years, this concept has been revamped by several studies. Few of the most recent ones have conveniently exploited proteomic technologies [12–14]. Indeed, it is not surprising that proteomic analysis will assist with elucidating new mechanisms of proteotoxicity as they happen not only in the brain but also in other organs, such as the heart. Of particular interest is the role of protein PTMs [15, 16]. These can be placed both enzymatically (such as phosphorylation) or occur as the result of environmental stress (such as oxidation). The latters are not regulated and therefore they can accumulate in a pathological fashion [12, 15]. If protein misfolding is a mechanism which can result in the uncontrolled accumulation of toxic species in the heart (such as PAOs), these technologies will greatly help in dissecting the relative contribution of different PTMs (chemical and enzymatic) to the etiology of several diseases, including HF.

### 2.4. What “Lies” Ahead

As new technologies and approaches become available to the medical community, it is challenging to remain up-to-date and pick those that will have a long-lasting impact. In this concluding paragraph three emerging methodologies which, in our opinion, are likely to play a major role in the future of clinical proteomics will be described briefly. The first is mass cytometry, made possible by the combination of flow cytometry and a TOF MS. In short this configuration enables to monitor several different antigens with little to no cross talk in a given cell subpopulation [17]. The second is MALDI imaging, combining the capabilities of MALDI-TOF MS with those of a microscope. Although its spatial resolution at this stage is limited, the distribution of a given peptide across a tissue section can be monitored with this approach [18]. Lastly, a new acquisition in the proteomic field is the possibility of accurately measuring the level of thousands of proteins at one time and retaining the capability of reinterrogating the obtained data with new questions that may arise even after the study is concluded. This novel approach has profound implications for clinical studies as it allows the creation of the* in silico* version of a proteome and its repeated interrogation. For example, this is particularly important for studies on HF due to the limited availability of (control) tissue [19]. The pioneering work of Aebersold and colleagues transferred the quantitative capability of MRM to proteome-wide approaches by using new acquisition methods. The increased speed of acquisition of certain MS configurations combined with “unbiased” detection approaches (Data Independent Acquisition or DIA) now permits “scanning” through a proteome by missing limited information. This information can be stored* in silico* and the resulting database can then be used as a reference to compare several different biological conditions and track back changes in a quantitative fashion. Several other applications are underway, including real-time diagnostics that could be particularly helpful in the operating room. With all these new technologies and knowledge there is an emerging need for technical expertise and education of the scientific community at large. The implementation of new software and algorithms to handle the amount of data that are being rapidly generated is an important task for the bioinformatic community. Lastly, local regulations will have to quickly adapt to allow the clinical community to translate efficiently the potential assays provided by these new tools into clinical practice.

## 3. Novel Medical Therapies in Heart Failure

### 3.1. Heart Failure with Reduced Ejection Fraction (HFrEF)

In the past 25 years, there have been substantial improvements in the treatment of patients with chronic HFrEF. This is also due to the increased availability of drugs acting on the renin-angiotensin-aldosterone system (RAAS) and on the adrenergic systems, such as ACE inhibitors (ACE-Is), angiotensin receptor blockers (ARBs), beta-blockers (BBs), and mineralocorticoid receptor antagonist (MRAs), proved by international randomized trials to be able to modify the natural history of this syndrome by prolonging survival [20].

Several demonstrations of a class effect of these drugs have been proven in the past years, and there is now a need for new drugs targeting different pathological pathways in order to further improve survival in HF patients. In a recent update from the European Society of Cardiology (ESC) guidelines on the management of acute and chronic HF, two major changes in the pharmacological treatment of patients with chronic HFrEF must be acknowledged:

- MRA treatment extension to patients with mild-to-moderate HFrEF as a consequence of the results of the EMPHASIS-HF trial (Eplerenone in Mild Patients Hospitalization and Survival Study in HF) [21], which enrolled 2737 patients aged ≥55 years with New York Heart Association (NYHA) functional class II symptoms and an ejection fraction (EF) <30% (<35% if the QRS duration was >130 ms). In this study, about 27% of relative risk reduction in cardiovascular death or HF hospitalization (primary outcome) was achieved with eplerenone treatment (up to 50 mg once daily). These results led to a class IA recommendation for MRAs in these guidelines.
- The SHIFT trial (Systolic Heart Failure Treatment with Ivabradine Compared with Placebo Trial) [22] has demonstrated that the use of ivabradine, a selective inhibitor of the If current in the sinoatrial node was associated with a significant reduction in the primary endpoint (cardiovascular disease related death or hospitalization for HF), mainly driven by a decrease in the rate of hospitalization in patients with NYHA class II or III HFrEF. This result is particularly relevant because it is the first drug to prove a clinically relevant result demonstrated in a randomized clinical trial in HFrEF patients by acting on pathophysiological systems different from the RAAS and the adrenergic system. Ivabradine was approved by the European Medicines Agency in 2012 for chronic HF in patients with elevated heart rates but at present it is not yet commercialized in the US.

Several new classes of drugs have been proposed or are under evaluation (Table 1):

- Finerenone (BAY 94-8862) is a next-generation nonsteroidal MRA that has shown improved selectivity for the mineralocorticoid receptor.
- Aliskiren, a direct renin inhibitor, decreases PRA and thus may provide a greater RAAS blockade.
- Omapatrilat is a molecule that was both a neprilysin and an ACE-I and whose development was terminated because of an unacceptable incidence of angioedema [23].
- Angiotensin receptor neprilysin inhibitors (ARNIs) are a new class of drugs developed both to block the RAAS and augment natriuretic peptides by the combination of an angiotensin II type 1 receptor blocker and an inhibitor of neprilysin, also known as neutral endopeptidase, the enzyme which promotes breakdown of atrial and brain natriuretic peptides (ANP and BNP, resp.).
- Riociguat is a novel soluble guanylate cyclase stimulator, which produces cGMP, the second messenger of several biologically active molecules such as nitric oxide or natriuretic peptides, and may improve central and peripheral hemodynamics.
- Darbepoetin Alfa, an erythropoiesis stimulating agent, and intravenous iron may improve outcomes in patients with HF and anemia, a common comorbidity in HF. Patients experiencing both conditions have a lower functional capacity, worse quality of life, and higher rates of hospitalization and death than those without anemia [24].
- Incretin based therapies have been developed in recent years to treat type 2 diabetes mellitus (DM). These agents include glucagon-like peptide-1 (GLP-1) agonists (exenatide and liraglutide) and dipeptidyl peptidase-4 inhibitors (sitagliptin, saxagliptin, and linagliptin). Animal and proof-of-concept clinical studies have shown cardioprotective effects of these drugs and potential benefits in patients with HF [25]. However, a large randomized clinical trial (SAVOR) testing the DPP4 saxagliptin showed a higher rate of occurrence of HF in patients with type 2 DM at high risk of CV events [26]. Another trial testing the DPP4 alogliptin (EXAMINE trial) showed no beneficial effect of the drug in patients with type 2 DM and a recent ACS [27]. With respect to GLP-1 agonist, large-scale clinical trials are still ongoing (EXCEL, ELIXA).

### 3.2. Heart Failure with Preserved Ejection Fraction (HFpEF)

“No treatment has yet been shown, convincingly, to reduce morbidity and mortality in patients with HFpEF.” This is the beginning of the very brief paragraph dedicated to pharmacological treatment of HFpEF in the ESC guidelines for the diagnosis and treatment of acute and chronic HF published in 2012. There is in fact substantial lack of evidence in the management of HFpEF patients: many of the treatments that have shown a benefit in HFrEF have failed to confirm their positive effects in patients with HFpEF. This is the case of* ad hoc* performed trials performed with ACE-I (PEPCHF) [28] and angiotensin receptor antagonists (CHARM-Preserved [29] and I-PRESERVE [30]).

Different reasons for the unsuccessful effects of these medications have been proposed: some were related to the patients (e.g., lack of specific symptoms with inappropriate enrollment and no agreement on the threshold of EF for definition of preserved systolic function), some were related to the trials (e.g., prolonged recruitment with a high rate of dropouts), some were related to the disease (e.g., different stages of disease), and some others were related to the tested drugs. For other classes of drugs such as BBs, we lack evidence from clinical trials (because no specific trial has ever been designed) and data derived from registries are quite controversial [31].

Since the publication of ESC guidelines, one large outcome trial (Treatment of Preserved Cardiac Function HF with an Aldosterone Antagonist, TOPCAT) and several proof-of-concept studies have been published where well accepted therapies in HFrEF and new therapeutic agents have been tested (Table 2).

The idea of evaluating the effect of drugs that have been proved to be of efficacy in patients with HFrEF in the setting of HFpEF has led to* TOPCAT*, the first international, multicenter, and randomized double-blind trial to assess the effect of spironolactone on clinical outcomes in the patients with HFpEF. It failed to demonstrate a significant improvement in the primary outcome, a composite outcome of cardiovascular mortality, aborted cardiac arrest, or HF hospitalization. These results were disappointing since the Aldosterone Receptor Blockade in Diastolic HF,* Aldo-DHF* trial had on the contrary given at least in part promising results. The substantially neutral results of TOPCAT may be explained by geographical differences in the characteristics of patients enrolled in some countries in which healthier patients were included (e.g., patients enrolled in the placebo group in Russia and Georgia experienced a significantly lower incidence of the primary endpoint compared to those in North or South America, 8.4 versus 31.8%) in which the treatment could not demonstrate a benefit, probably resulting in a dilution of the global effect.

These disappointing results have given the impulse for research in individualizing new therapeutic targets by exploring different pathophysiological pathways such as the nitric oxide (NO) myocardial cyclic guanosine 3′,5′-monophosphate-protein kinase-G pathway (NO-cGMP-PKG). At least some of the beneficial effects induced by NO and natriuretic peptides are in fact mediated by stimulation of soluble and membrane-bound guanylate cyclases, respectively, which produce the second messenger cGMP. Phosphodiesterase-5 (PDE-5) metabolizes cGMP and may limit beneficial NO and natriuretic peptide actions and reduce cGMP-mediated improvements in myocardial relaxation and hypertrophy reduction. The hypothesis that the PDE-5 inhibitor sildenafil might have some benefits in patients with HFpEF was tested in the* RELAX* trial.

The* PARAMOUNT* trial targeted this pathway from a different point of view and tested in patients with HFpEF LCZ696, a first-in-class ARNI that is a complex molecule from the combination of the neprilysin inhibitor prodrug AHU377 and the ARB valsartan. Neprilysin degrades biologically active natriuretic peptides which, as described above, stimulate the production of cGMP.

Inflammation seems to have an important role in the pathophysiology of HFpEF. The* D-HART* pilot study was a small double-blind, randomized, placebo-controlled, and crossover trial that tested anakinra, an interleukin-1 inhibitor, in patients with HFpEF. Anakinra led to a statistically significant improvement in the primary endpoint, which was peak oxygen consumption (+1.2 mL/kg/min, *p* = 0.009), and a significant reduction in plasma C-reactive protein (CRP) levels (−74%, *p* = 0.006). The reduction in CRP levels correlated with the improvement in peak oxygen consumption (*R* = −0.60, *p* = 0.002). Impaired relaxation is a fundamental component of HFpEF and for this reason there is a strong pathophysiological rationale for the utilization of a drug like ranolazine in this setting. The* RALI-DHF* (RAnoLazIne for the Treatment of Diastolic HF) study was a prospective, randomized, double-blind, placebo- controlled, small, and proof-of-concept study.

## 4. Surgical Alternatives in the Therapy of Severe Left Ventricular Dysfunction

### 4.1. The Role of Coronary Revascularization

The most common cause of heart failure with severely depressed left ventricular ejection fraction (LVEF) is ischemic heart disease, accounting for more than 60% of cases [32]. Ischemic etiology of left ventricular (LV) systolic dysfunction leads to significantly higher mortality rates than other etiologies [33]. The explanation of this aggressive course is the well-known relationship among myocardial ischemia, interstitial fibrosis, and endothelial dysfunction, often with associated systemic comorbidities, such as diabetes, which worsen the natural history.

Observational studies comparing survival in patients treated surgically versus medically suggested that coronary artery bypass grafting (CABG) enhances survival in patients with ischemic cardiomyopathy [34–41]. Significant reductions from >50% to 10% in mortality have been demonstrated with surgery if compared with medical therapy.

Results reported from early trials comparing medical therapy with CABG for the treatment of stable angina have a limited value in the current era because both surgical techniques and medical therapy have significantly and rapidly improved. Arterial grafts were rarely used in these trials, and medical therapy largely consisted of only nitrates and infrequent use of beta-blockers. Finally, patients with severe LV dysfunction were largely excluded from the enrollment. However, the Veteran Affairs Cooperative Study of Surgery [42] and the Coronary Artery Surgery Study [43] demonstrated a significantly higher survival rate in the patients with reduced LVEF after CABG in comparison with those who were randomized to medical therapy. Other studies confirmed that CABG in patients with severely depressed LVEF obtained a satisfactory survival rate similar to cardiac transplantation [44, 45].

Several contemporary trials studying treatments for ischemic coronary disease that included an intensive medical therapy, such as the MASS-II (Medicine, Angioplasty, or Surgery Study) trial and the COURAGE (Clinical Outcomes Utilizing Revascularization and Aggressive Drug Evaluation) trial, excluded patients with severe LV dysfunction [46, 47]. The BARI 2D (Bypass Angioplasty Revascularization Investigation in Type 2 Diabetes) trial included patients with LV dysfunction but only 17.5% of patients had LVEF <50% [48]. The ISCHEMIA (International Study of Comparative Health Effectiveness with Medical and Invasive Approaches) trial is currently enrolling patients but exclusion criteria include LVEF <35% [49].

Among 27 randomized controlled trials comparing CABG and percutaneous coronary intervention (PCI) [50], most of the patients had preserved LV systolic function (EF >50%). None of these trials specifically focused on patients affected by heart failure and/or LV systolic dysfunction. Two relatively large trials that included patients with depressed LVEF were BARI (Bypass Angioplasty Revascularization Investigation) [51], in which 22% of patients enrolled had LVEF <50%, and AWESOME (Angina with Extremely Serious Operative Mortality Evaluation) [52], in which 21% had LVEF <35%. Analyses of this subset of patients from these trials confirmed no differences in outcome between PCI and CABG [45, 53]. Moreover, the most recent trials comparing PCI with CABG failed to provide a clear superiority. The SYNTAX trial enrolled approximately 2% of patients with LVEF <30% [54]. The FREEDOM trial [55] reported similar outcomes with PCI with drug-eluting stents and CABG in patients with LVEF <40%, but only 2.5% of the patients were in this prespecified subgroup with depressed LVEF. Thus, the available data have insufficient statistical power to adequately compare PCI and CABG in patients with severe LV dysfunction.

The STICH trial is the only prospective, randomized, and controlled trial designed to study the role of CABG in patients with LVEF ≤35%. The aim of this trial was to test 2 hypotheses among patients with LVEF ≤35% and CAD amenable to CABG [56]: the comparison between CABG and medical therapy (MT) alone in 1,212 patients and the surgical ventricular restoration (SVR) hypothesis compared CABG with and without SVR in 1,000 patients. In the intention-to-treat analysis, no significant difference was observed in the primary outcome of all-cause mortality between patients randomized to CABG versus MT over a median follow-up period of 56 months. The CABG group reported improved rates of death from cardiovascular causes and lower rates of a combined endpoint of death from any cause and hospitalization for heart failure, which were secondary endpoints of the study [56]. Moreover, as-treated and adjusted analyses to consider patient crossovers suggested an overall favorable effect of CABG on primary and secondary outcomes [57, 58]. Final data derived from this trial suggest that the observed survival benefits of CABG in patients with severe LV dysfunction are related primarily to factors such as functional status assessed by a 6 min walk and/or the Kansas City Cardiomyopathy Questionnaire [59] and the interaction of angiographic severity of CAD, severity of LV systolic dysfunction, and severity of LV remodeling [60]. Patients with preserved effort tolerance but with multivessel CAD, lower EF, and higher end-systolic volume index were most likely to benefit from CABG, particularly with respect to long-term survival.

### 4.2. Surgical Ventricular Reconstruction

Changes in LV structure and function secondary to heart failure include remodeling of the LV from its normal elliptical shape to a more spherical shape. These modifications result in a dysfunctional, less efficient, and low contractile ventricle and are predictors of worse prognoses. Surgical ventricular reconstruction (SVR) may potentially reverse this process and partially restore functional capacity of LV [61–63]. Vincent Dor described in 1989 a technique of endoventricular circular patch plasty more commonly known as surgical ventricular restoration or Dor Procedure [64]. This technique consists of aneurysm resection with insertion of a circular Dacron or pericardial patch to reconstruct the ventricle. Surgical restoration therefore excludes akinetic septal regions of the LV and restores LV chamber size and shape to more physiologic conditions. Associated CABG showed significantly improved LV function and outcomes at 1 year [65]. The beneficial effects of SVR were further confirmed by the publication of outcomes of the Reconstructive Endoventricular Surgery returning Torsion Original Radius Elliptical shape to the LV (RESTORE) Group. In this study, 1,198 post-MI patients with HF were treated, showing an overall 30-day survival of 94% and 5-year survival of 69% [61].

However, it remained uncertain whether SVR combined with CABG would result in improved outcomes of patients with ischemic cardiomyopathy compared with CABG alone, particularly when associated with optimal medical therapy. This question led to the surgical ventricular reconstruction arm of the STICH trial [66]. In this arm, patients were enrolled if they had coronary disease amenable to surgical revascularization, severe systolic dysfunction with LVEF ≤35%, and significant LV anterior akinesia or dyskinesia that was amenable to SVR. A total of 1,000 patients were randomized to CABG alone versus CABG plus SVR. The primary outcome was a composite of all-cause mortality and cardiac hospitalization. The study showed no significant difference between the 2 therapies for the primary outcome with a median follow-up of 4 years. There were also no differences between the 2 groups in terms of secondary endpoints, including repeat hospitalizations, symptoms, or quality of life [66]. SVR associated with CABG does not appear to improve quality of life compared with CABG alone but does increase health care costs [67].

**Figure 1:**
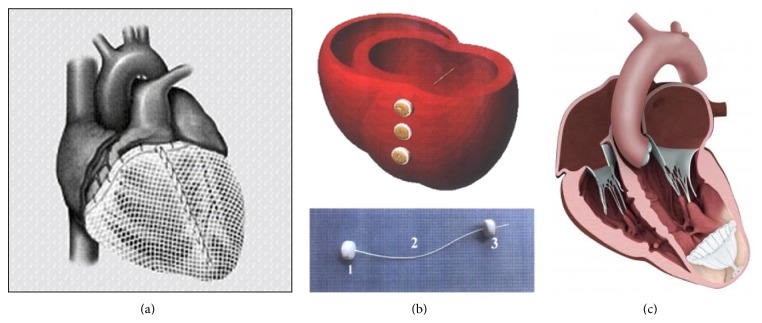
(a) Acorn CorCap Cardiac Support Device. (b) Myosplint system. (c) Parachute ventricular partitioning device.

In the last years, several devices designed to restore LV geometry and decrease wall stress have been evaluated. The most tested has been the Acorn CorCap Cardiac Support Device (Acorn Cardiovascular, Inc). This device consists of a polyester mesh sutured circumferentially around the heart from the apex to the atrioventricular groove (Figure 1(a)). It provides circumferential support, decreases LV wall stress, and avoids progressive chamber dilatation [68]. The results of the pivotal Acorn clinical trial have been already published. Three hundred patients affected by HF were randomized to CorCap implantation with mitral surgery versus mitral surgery alone and to CorCap plus medical therapy versus medical therapy alone. Totally, 148 patients received CorCap: they demonstrated that they need less subsequent procedures (Cardiac Resynchronization Therapy or CRT, CABG, and repeat mitral surgery), an improvement in NYHA class and Minnesota Living with Heart Failure score (MLHF), and favorable echocardiographic reverse remodeling. However, no improvement in survival could be demonstrated at 1, 3, or 5 years [69–71].

The Myosplint system utilized three tensioning rods placed transversely through the left ventricle at the apex, mid, and base, respectively, secured and tensioned by epicardial pads. This device was designed to create a bilobular LV cross section with the aim of reducing the radius of the chamber of LV and consequently its wall stress (Figure 1(b)). The device was tested in 21 consecutive patients to demonstrate safety and feasibility. Whereas the original concept was proven safe and feasible, the authors concluded that the device did not address a satisfactory functional mitral regurgitation repair as the majority of patients who received the Myosplint also needed mitral valve surgery [72].

Coapsys device consisted of a similar tensioning device which bisected the heart and was connected by anterior and posterior epicardial pads. The aim of this device was to reposition the papillary muscles by being placed at the level of the mitral subvalvular apparatus and approximating the ventricular walls. It could be placed through sternotomy without cardiopulmonary bypass [73]. A large multicenter randomized study, the Randomized Evaluation of a Surgical Treatment for Off-Pump Repair of the Mitral Valve (Restor-MV trial), enrolled patients with CAD and functional MR to two arms: CABG with MV repair and CABG alone. The former arm was further randomized to CABG with traditional annuloplasty versus CABG with Coapsys. The latter arm, treated with CABG alone, was further randomized to CABG alone versus CABG with Coapsys.

The trial was stopped in advance because the researchers failed to secure funding for its continuation. Nevertheless, the study demonstrated a survival advantage as well as decreased adverse events at 2 years in patients treated with CABG and Coapsys compared to those treated with CABG and standard annuloplasty [74].

The concept of a less invasive procedure to obtain LV restoration was exploited by the company BioVentrix, which developed the Less Invasive Ventricular Enhancement (LIVE) therapy utilizing the Revivent technology. The technique involves beating heart isolation of scarred and akinetic myocardium by using the Revivent Myocardial Anchoring System. This system is then placed by epicardial perforation of the myocardium and interventricular septum (IVS) at the borders of the scarred area. The internal anchor is then inserted inside the RV and anchored at the right ventricular surface of the IVS. Consequently, the LV lateral and IVS walls are slowly and completely apposed and the scar is isolated. Further investigations as to the role of this device are ongoing in a phase II study.

In 2006, Sharkey and colleagues described a left ventricular apex occluder, named ventricular partitioning device (VPD) [75]. The system components are three: an access system, a delivery system, and the VDP. The access system is comprised of a 14–16 F guide catheter and dilator which provide access to the apical LV. The catheter is used to deliver the collapsed VPD through the aortic valve to the apex of the LV. The delivery system has an inner lumen with a balloon just proximal to the engagement screw, which is used to inflate the VPD in order to achieve the anchoring of its struts against the ventricular walls, ensuring adequate isolation of the LV apex and stability of the device. The VPD consists of an expanded polytetrafluoroethylene (ePTFE) occlusive membrane associated with a self-expanding Nitinol frame shaped like an inverted umbrella or parachute with 16 struts. This device separates the enlarged and scarred left ventricle into two chambers: one dynamic and one static. The static chamber is the scarred or aneurysmal part of the LV that is distal to the device hemodynamically isolated by the occlusive device membrane; the dynamic chamber is the remaining normal LV (Figure 1(c)). This division causes regional hemodynamic unloading of the isolated dilated apical left ventricle, decreasing wall stress in that region. Moreover, the dynamic LV becomes less voluminous with partitioning, leading to volume/pressure unloading of the functioning myocardium [76]. The first human trial with the Parachute device (CardioKinetix Inc, Menlo Park, CA) was a single arm, prospective, and nonrandomized multicenter study that enrolled 39 patients. The primary end point was technical safety of the device as well as device-related complications within the first 6 months of follow-up. Inclusion criteria were anteroapical akinesis from an anterior myocardial infarction, LVEF of ≤40%, advanced NYHA class, and stable optimal medical therapy for at least 3 months before enrollment. Exclusion criteria were ischemic CAD requiring revascularization, previous revascularization, or CRT within 60 days and patients with significant valve disease. Of the 39 patients, five were thought to have unsuitable LV anatomy for VPD placement after enrollment. After this, the protocol was changed to include computed tomographic (CT) evaluation before enrollment to determine LV anatomical suitability. VPD was successfully delivered in 79% of patients initially enrolled and in 91% of patients in whom it was technically attempted. Overall 6-month success rate without events related to the device was 74%. Hemodynamically, despite significant reductions in LV volumes, LVEF and stroke volume index remained unchanged. Nevertheless, there was a significant decrease in NYHA class, and there were trends towards improvement in QOL measure and 6MWD, although they were not statistically significant [77].

Therefore, Parachute has encouraged with widespread enthusiasm the design of four trials in different stages of completion: the PARACHUTE Trial cohorts A and B following 89 patients enrolled, the PARACHUTE US trial enrolling 20 patients in 8 USA institutions, the PARACHUTE III postmarketing trial in 20 European centers to follow-up 100 patients, and the PARACHUTE IV trial that has begun patients enrollment in the second quarter of 2012 with the aim to enroll 478 patients across 65 USA institutions.

### 4.3. Mitral Valve Surgery

Functional mitral regurgitation (FMR) is a pathological condition resulting from geometrical distortion of the subvalvular apparatus secondary to LV enlargement and remodeling and due to idiopathic or ischemic cardiomyopathy [78]. Thus, FMR is not a primary mitral valve disease but the result of the previously mentioned complex remodeling processes of LV; however, its presence leads to further remodeling [78]. Surgeons have tried in the last decades to find the specific solutions to this topic with contradictory results and unresolved answers.

While some studies on FMR correction are ongoing, to date no data have clearly demonstrated the superiority of surgery versus optimal medical therapy [78–82]. Results from the Michigan University study showed that no evident advantage is obtained by mitral valve (MV) annuloplasty when compared to optimal medical therapy in terms of 5-year event-free survival in patients with mitral regurgitation and left ventricular systolic dysfunction of any origin; moreover, the same results were reached comparing optimal medical therapy to mitral valve annuloplasty for patients with nonischemic etiology of FMR and LV dysfunction [83]. Another study by Kang et al. similarly showed that MV annuloplasty plus CABG in ischemic cardiomyopathy did not show any 5-year survival advantage when compared to isolated CABG alone but carried the weight of a higher operative mortality due to concomitant mitral surgery [84]. However, the same study showed a clear advantage by adding MV surgery in terms of residual mitral regurgitation in the follow-up of those patients suffering from severe grade of ischemic FMR [84]. Wong et al. [85] found that mitral annuloplasty in patients with moderate MR did not improve 1-, 5-, and 10-year survival but only the corresponding degrees of residual valvular regurgitation.

Other authors reported similar 5-year survival between isolated CABG and CABG plus MV surgery despite a significant lower mitral regurgitation (MR) grade at 1 year and a trend toward a lower MR grade at 5 years in the second group [86]. The same authors confirmed in a propensity-matched analysis a similar 1- and 5-year survival and functional class with both surgical procedures (CABG versus CABG plus MV repair) but a significant reduction in MR grade at 1 year by adding MV repair [87].

On the other hand, some literature data support the benefit of surgery for FMR. Trichon and coworkers analyzed a wide series of patients with ischemic FMR and found that PCI, CABG, and CABG plus MV repair all obtained a 3-year survival advantage compared to medical therapy in this subset of patients [88]. A retrospective study from the Brigham and Women's Hospital in Boston recently reported an improved survival of patients undergoing mitral valve repair for cardiomyopathy [89]. A recent analysis of more than 1,200 patients enrolled in the STICH Trial for ischemic cardiomyopathy demonstrated that concomitant MV repair in patients with moderate-to-severe FMR who underwent CABG reduces 30-day mortality compared to either patients undergoing isolated CABG or those medically treated [90]. Furthermore, these results were confirmed when 5-year survival was analyzed in the same subgroups of patients [90]. The recently published results from the RIME trial, a randomized controlled multicenter study from United Kingdom, showed that addition of MV surgery to CABG in the setting of ischemic cardiomyopathy resulted in similar 1-year survival, rate of hospital admission, and recurrence of atrial fibrillation; on the other hand, the study demonstrated greater 1-year improvement in the primary end point of peak oxygen consumption in the CABG plus MV repair group compared with the CABG group with a better LV reverse remodeling and a higher reduction in MR grade and in serum BNP values [91].

When nonischemic cardiomyopathy was considered, despite the absence of randomized controlled trials comparing surgery with optimal medical therapy and different techniques of surgery and despite the lack of data of a clear survival advantage potentially obtained with the correction of the functional incompetent mitral valve, recent literature studies all confirmed the beneficial impact of surgery [92–95].

Finally, percutaneous mitral valve therapies are of particular interest in patients at high risk for surgical intervention, including those with secondary MR related to CAD and heart failure. Promising results have been reported from Europe in such patients who remain symptomatic despite optimal medical therapy and CRT [96–98]. Two ongoing randomized trials of transcatheter valve repair versus medical management [99, 100] may clarify whether treating the mitral valve in addition to optimal medical therapy improves outcomes of patients with ischemic MR.

## 5. New System or New Materials Available for Future Cardiac Assist Devices

**Figure 2:**
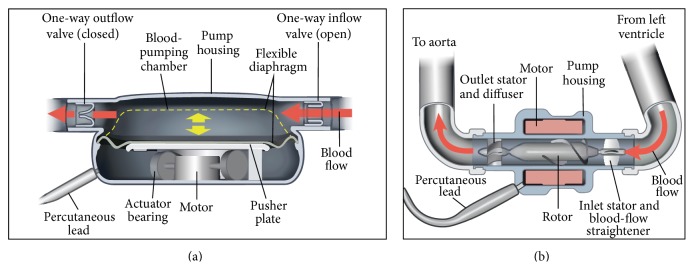
(a) Pulsatile pump. (b) Continuous flow pump.

The expansion of cardiac transplant centers without any increase in donor supply led to longer waiting lists and longer time to transplantation, during which the prolonged benefit of the LVAD to provide support for over a year became apparent, although the majority of patients required support for shorter periods [101]. The overall survival to transplant after LVAD support has been around 65% during the past 5 years [102]. The next advance was the demonstration that the LVAD could also double survival as permanent “destination” therapy in patients not eligible for transplant. All these devices come hand in hand with a heavy medication, especially anticoagulants, creating a new weakness. The need of an external, anticoagulant-free, and ventricular assistance as a bridge to decision and/or permanent “destination” therapy is indubitable. A growing number of heart devices and machines are being used in heart failure treatment [103–107]. Ventricular assist devices (VADs) are machines that help improve pumping. They have gained well-known approval for use as a bridge to transplant in patients who are on medications but still have severe symptoms and are awaiting a donor heart. Nevertheless, more and more doctors are exploring the possibility that such devices may be adequate treatments themselves, preventing the need for a transplant in some patients. Therefore, they may be used as short-term (less than 1 week) or longer-term support [102, 108–114]. Most of the devices that we are using make an intrusion in the body and more critically in the heart (Figure 2). They are in direct contact with blood and need high doses of anticoagulants to function properly. Recently, we have studied a revolutionary system that seems to address all these drawbacks. The key advantages of this new BiVAD are the biventricular assistance and its external positioning. This last point improves first of all the operative ease and security. Second, there is no direct contact with circulating blood getting rid of anticoagulants and furthermore it lowers the probability of rejection. An additional benefit is the possibility of differential assistance. In other words, this BiVAD is adjustable to any heart presenting left, right, or both sided dilatation [111, 112]. The tool mainly involved in the development of this new assist device is the metal alloy of nickel and titanium (Ni-Ti) Ninitol, where the two elements are present in roughly equal amount. Small changes in composition can significantly impact its properties. This alloy exhibits two closely related and unique properties: shape memory and superelasticity [115–118]. Shape Memory Alloys (SMAs) are a group of metallic materials that demonstrate the ability to return to some previously defined shape or size when subjected to the appropriate thermal procedure [118]. They have an austenitic (“hot”) phase in which the material is generally stiffer and has a higher yield point, and a martensitic (“cool”) phase which is less stiff and has a lower yield strength. In the low temperature, crystal phase they are generally superelastic. This means they can be deformed far more than other metals (approx. 10–20 times) of the same general family. They can be formed into a shape at higher temperature, cooled, and then formed to a different shape at room temperature. When heated, they return to the shape they had at the higher temperature. This may be repeated through several million cycles. There are several known metal combinations that have these properties. Nickel-Titanium (Ni-Ti or Nitinol) has proven to be the most flexible and useful SMA in engineering applications so far. It has greater ductability, more recoverable motion, excellent corrosion resistance, stable transition temperatures, high biocompatibility, and the ability to be electrically heated for shape memory recovery.

**Figure 3:**
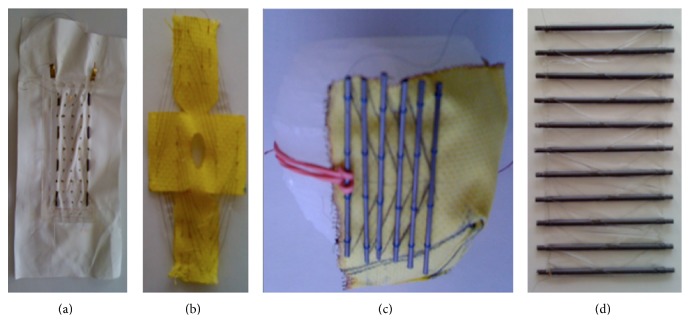
Four different methods to weave Nitinol. From left to right: (a) Nitinol woven on PTFE with a solid U-shaped structure; (b) Nitinol woven on Kevlar in a circular manner; (c) Nitinol woven around a carbon-tube structure fixed on a Kevlar envelope; (d) silicon matrix with carbon tubes and Nitinol weaning weaving.

**Figure 4 fig4:**
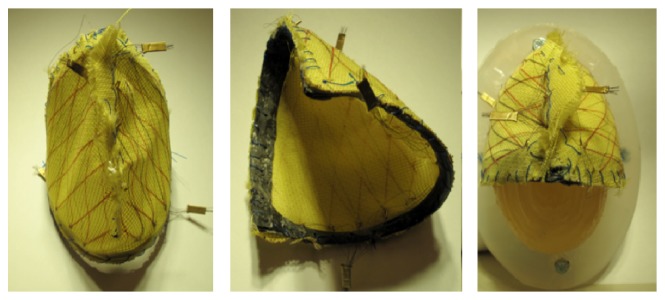
Ventricular “envelope” with a Nitinol woven structure on both sides.

**Figure 5:**
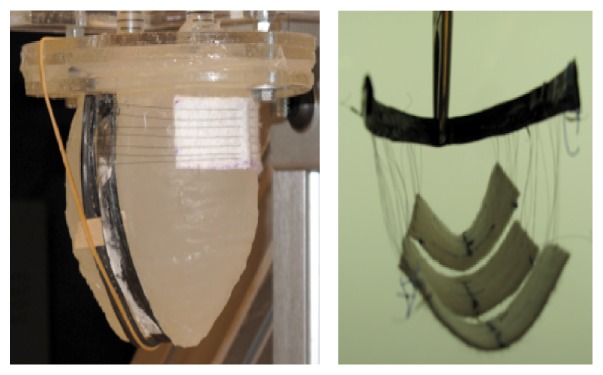
Wires around the ventricle with a slice of PTFE between the wall and the wires.

Several designs are under study [119–123]. One of them is based on a configuration where the Nitinol wires could be weaved on a tissue or a membrane that would be in direct contact with the heart walls. Besides, the wires deliver the highest force when weaved with an angle of 20° between each other. There are many ways to arrange a wire with this angle; one of those was an accordion like structure weaved on Kevlar or Teflon (polytetrafluoroethylene, PTFE). Another configuration is to realize a spiraling pattern keeping the idea of the 20° angle. Lastly, another solution is to use a solid structure surrounding the heart between each ventricle (Figure 2). Thanks to this carbon structure, the Nitinol wires can be attached and pulled until they are tightened around each ventricle. It is now possible to have different configurations surrounding each ventricle. In addition to that, two different wire lengths were tried. Either there was a unique wire going several times from one side of the structure to the other or there were many wires for each come and go (Figures 3 [Fig fig4]–5).

## 6. Conclusions

The modern approach to the diagnosis and treatment of heart failure is multidisciplinary and should be based on a close collaboration among researchers, clinicians, and cardiac surgeons, particularly given that mandatory multiorgan attention is required in these high risk patients.

Future therapies for heart failure could include ventricular assist devices implantation or ventricular restoration techniques with the aim to obtain a reverse, positive remodeling in the unloaded heart.

With an expanding “toolbox” of comprehensive basic, medical, surgical and technological approaches, it is expected that these novel findings will soon be translated to the clinical practice. In fact, new therapeutic strategies are desperately needed by the millions of patients suffering from heart failure.

## Figures and Tables

**Table 1:** Recent studies in heart failure with reduced ejection fraction.

Study	Type	Drug/comp.	Number of pts/Age	Outcome	Results
ARS	PoC	Finerenone (BAY 94-8862) (nonsteroidal MRA)	458/72	Safety and tolerability in chronic kidney disease versus spironolactone	Significantly lower incidences of hyperkalemia than spironolactone

ATMOSPHERE	Outcome	Aliskiren (direct renin inhibitors)	7000	Cardiovascular death or HF hospitalization versus enalapril	Ongoing

LEPTH	Outcome	Riociguat (guanylate cyclase stimulator)	201/59	Change in mean pulmonary artery pressure	Not met but improved stroke volume and cardiac index and reduced pulmonary and systemic vascular resistance

SOCRATES-REDUCED	PoC	Vericiguat (guanylate cyclase stimulator)	410	Change in NT-proBNP	Ongoing

PARADIGM-HF	Outcome	LCZ696 (ARNI, angiotensin II receptor blocker and neprilysin inhibitor)	8442/63	Death from cardiovascular causes or a first hospitalization for heart failure	LCZ696 was superior to enalapril in reducing the risks of death and of hospitalization for heart failure

RED-HF	Outcome	Darbepoetin Alfa	2278/72	Death or hospitalization in patients with Hb (9.0–12.0 g/dL)	Not met but improved haemoglobin level

FAIR-HF	PoC	Intravenous iron (ferric carboxymaltose)	459/67	Self-reported Patient Global Assessment and NYHA functional class	Improvements in 6-minute walk test and quality of life assessment

ICHF	PoC	Intravenous iron (ferric carboxymaltose)		Improvement in LVEF	Ongoing

MOOD-HF	Outcome	Escitalopram (serotonin reuptake inhibitor)	700	Death or hospitalization	Ongoing

PoC, proof-of-concept.

**Table 2:** Recent and ongoing studies in heart failure with preserved ejection fraction.

Study	Type	Drug/comp.	Number of pts/Age	Outcome	Results
TOP-CAT	Outcome	Spironolactone versus placebo	3445/69	Primary outcome: CV death/HF hospitalization/aborted cardiac arrest	In follow-up 3.3 years 18 versus 20 (*p* = 0.14)

Aldo-DHF	PoC	Spironolactone versus placebo	422/67	Coprimary outcomes: (i) Diastolic dysfunction (E/E′) (ii) Exercise capacity/peak VO2	In 12-month follow-up (i) 12.1 versus 13.6 (*p* < 0.001) (ii) 16.8 versus 16.9 (*p* = NS)

RELAX	PoC	Sildenafil versus placebo	216/69	Primary outcome: exercise capacity/peak VO2 Secondary outcome: (i) 6 min walk test (ii) Clinical outcome	In 24-week follow-up (i) −0.2 versus −0.2 (*p* = NS) (ii) 5.0 versus 15 m (*p* = NS) (iii) 94 versus 95 (*p* = NS)

PARAMOUNT	PoC	LCZ 696 angiotensin rec. + Neprilysin inhib. versus valsartan	266/71	Change NT-proBNP Side effects	Ratio LCZ696/valsartan 0.77 (*p* = 0.005) 22 patients (15%) on LCZ696 versus 30 (20%) on valsartan

DHART	PoC	Anakinra versus placebo	12/62	Exercise capacity/peak VO2	+1.2 mL/kg/min (+8%, *p* = 0.009)

RALI-DHF	PoC	Ranolazine (iv 24 h infusion followed by 13 days of oral treatment) versus placebo	20/73	Changes in hemodynamic parameters Changes in echocardiography, PeakVO2, and NT-proBNP parameters	LVEDP (mmHg) 23 versus 19 (*p* = 0.04); PCWP 18 versus 12 (*p* = 0.04) No changes (*p* = NS)

Kosmala	PoC	Ivabradine versus placebo	61/67	Exercise capacity (METS) Peak VO2	+1.5 versus +0.4 (*p* = 0.001) +3.0 versus +0.4 (*p* = 0.003)

PARAGON-HF	Outcome	LCZ956 versus valsartan		CV death and HF Hospitalization	Ongoing

SOCRATES-PRESERVED	PoC	Vericiguat (guanylate cyclase stimulator)		Change in NT-proBNP	Ongoing

EDIFY	Outcome	Ivabradine versus placebo	400	Diastolic dysfunction (E/E′, exercise capacity, NT-proBNP)	Ongoing

PoC, proof-of-concept.
